# Management of cardiogenic shock complicating acute myocardial infarction: A review

**DOI:** 10.1002/clc.23168

**Published:** 2019-03-27

**Authors:** Ashish H. Shah, Rishi Puri, Ankur Kalra

**Affiliations:** ^1^ St Boniface Hospital and University of Manitoba Winnipeg Manitoba Canada; ^2^ Cleveland Clinic Foundation Cleveland Ohio

**Keywords:** acute myocardial infarction, cardiogenic shock and management

## Abstract

Despite advances in percutaneous coronary interventions and their widespread use, mortality in patients presenting with acute myocardial infarction (MI) complicated by cardiogenic shock (CS) has remained very high, and treatment options are limited. Limited evidences exist, supporting many of the routinely used therapies in treating these patients. In the present article, we discuss CS complicating MI in general and an update on the currently available treatment options, including inotropes and vasopressor, coronary revascularization, mechanical circulatory support devices, mechanical complications, and long‐term outcomes.

## 1 INTRODUCTION

Cardiogenic shock (CS) continues to be the leading cause of mortality in patients presenting with acute myocardial infarction (AMI),^1^ the incidence ranging between 5% and 8%.^2^ Although with advances in treatment, mainly early revascularization, the overall mortality in patients presenting with AMI has markedly reduced, but still the mortality in patients presenting with AMI complicated by CS remains very high (≅50%).^1^ Limited evidences exist, supporting many of the routinely used therapies in treating these patients. The purpose of the present article is to highlight and discuss the significance of CS and presently available treatment options.

## 2 DEFINITION

CS is a state of decreased cardiac output resulting in end‐organ hypoperfusion in the absence of intravascular hypovolemia. Prior studies defined CS, using the markers of cardiac output and tissue perfusion, obtained either invasively, clinically, or biochemically.^3^ However, as per the **SH**ould we emergently revascularize **O**ccluded **C**oronaries for cardiogenic shoc**K** (SHOCK) trial, CS should be defined as^1^: persistent hypotension (systolic blood pressure <90 mm Hg, or requirement of vasopressor to maintain systolic pressure >90 mm Hg),^2^ reduction in cardiac output (CO) (<1.8 L/min/m^2^ without support or 2.0 to 2.2 L/min/m^2^ with support), in presence of elevated left ventricular end‐diastolic pressure (EDP).^3^ Clinically, signs of organ hypoperfusion, for example, cold extremities, reduced urine output, and altered mental status in extreme cases are present in these patients,^4^ as described in Table 1. Although the staging of shock has not been well defined, higher mortality was noted in patients who required inotropic agents, especially in higher doses.^5^

**Table 1:** Definition and signs of cardiogenic shock

Hemodynamic criteria
1. Systolic blood pressure (SBP) of less than 90 mm Hg for >30 minutes, or use of vasopressors/inotropes to maintain SBP greater than 90 mm Hg
2. Reduced cardiac output (<1.8 L/min/m^2^), or 2.0‐2.2 L/min/m^2^ with vasopressor/inotropic support, in presence of elevated pulmonary capillary wedge pressure
Signs of tissue hypoperfusion
1. Tachycardia
2. Pale, cool, and clammy peripheries, prolonged capillary refill time
3. Oliguria
4. Altered mental status/confusion
5. Elevated lactate
6. Mixed venous saturation of less than 65%

## 3 PATHOPHYSIOLOGY

Acute deterioration in the left ventricular (LV) contractility is usually the main cause of CS. However, impaired right ventricular (RV) systolic function and deranged vasculature functionality may also contribute toward establishment and/or worsening of CS. Reduced CO affects coronary perfusion, resulting in a downward spiral of impaired myocardial contractility and overall worsening of CS. The presence of obstructive atherosclerotic coronary artery disease may further exacerbate reduced coronary perfusion. Although left ventricular ejection fraction is a prognostic marker in patients presenting with CS,^6^ contrary to the general belief, LV systolic function is not always severely impaired.^7^ The observed left ventricular ejection fraction in the SHOCK trial was ≅30%, the value commonly noted in many of the trials evaluating heart failure and post‐myocardial infarction (MI) therapies.^4^, ^8^, ^9^, ^10^ Not only systolic impairment, but also diastolic dysfunction and associated restrictive filling pattern are common findings in patients presenting with CS.^11^ Similarly, isolated RV dysfunction can also result in CS, albeit in a very small number of patients (~5%), whereas in the majority of patients it co‐exists with LV dysfunction.^12^ RV dysfunction influences LV contractility not only by reducing LV preload, but also by influencing ventricular interdependence or by leftward bowing of the interventricular septum‐mediated change in the LV geometry and resultant contractility.^12^ In a small proportion of patients, ischemia also affects right atrial function, and results in reduced RV filling.^13^ Generally, patients presenting with CS due to RV dysfunction are younger, have less multivessel disease, and are less likely to have a previous history of MI.^14^ CS due to predominant RV failure has a similar mortality rate to that due to LV dysfunction.^12^ Some of the patients with CS may present with out‐of‐hospital cardiac arrest that is independently associated with very high in‐hospital mortality.^15^ Coronary artery disease is a major cause of cardiac arrest; either due to its presentation with AMI or ischemia induced ventricular tachyarrhythmias.

Hypoperfusion of vital organs triggers catecholamine and vasopressin release, aiming to improve end‐organ perfusion by augmenting myocardial contractility and peripheral vasoconstriction. In the short‐term, such neurohormonal changes improve tissue perfusion. However, persistently elevated levels have a detrimental effect on myocardial function due to elevated afterload and myocardial oxygen demand, especially in the context of reduced CO and impaired coronary perfusion. In the presence of neurohormonal activation, one would expect the systemic vascular resistance (SVR) to be elevated; the mean SVR in the SHOCK trial was in the normal range, and interestingly, 54/302 (18%) of the study cohort were suspected to have septic shock due to fever, leukocytosis, and significantly lower SVR.^16^

Up‐regulation of inducible nitric oxide synthase (i‐NOS), in response to inflammatory stimuli produces pathological amounts of nitric oxide (NO) that can promote inappropriate vasodilation along with inhibition of myocardial inotropy. Experimental animal studies demonstrated beneficial effects of selective iNOS blockade.^17^ Similarly, single‐center experience with this non‐selective NOS inhibition demonstrated promising results in patients with refractory CS.^18^ Whereas, such an approach failed to demonstrate mortality benefit, when compared with placebo in a randomized study.^19^ Similarly, elevated levels of other inflammatory markers, such as C‐reactive protein (CRP), tumor necrosis factor‐α (TNF‐α), interleukin‐6, and their subsequent change predicted death and occurrence of CS in patients presenting with STEMI.^20^ However, inhibiting complement component (C5), a downstream signaling of many inflammatory pathways, with pexelizumab failed to demonstrate beneficial effects either in the development of shock or associated mortality.^21^

In addition to ventricular dysfunction, mechanical complications of AMI, such as ventricular septal defect, free wall rupture, papillary muscle rupture can also result in CS. They contributed 12% of CS cases in the SHOCK trial.^22^ Although the rate of such complications has reduced since introduction of primary percutaneous coronary intervention (PPCI),^23^ their occurrence carries high mortality. Similarly, acute mitral regurgitation, either due to papillary muscle/chordae rupture or poor coaptation due to LV dilatation complicates AMI, resulting in CS. Such mechanical complications should be suspected, especially when patients present in CS with relatively small infarct size. Etiologies resulting in CS are described in Table 2, whereas risk factors associated with establishment of CS are listed in Table 3.

**Table 2:** Mechanisms of cardiogenic shock

Causes of CS associated with AMI
AMI without mechanical complications:
1. Severe left ventricular dysfunction (new ± pre‐existing dysfunction)
2. Severe right ventricular dysfunction (with/without LV dysfunction)
3. Arrhythmias secondary to ischemia
AMI with mechanical complications:
1. Papillary muscle or chordal rupture, resulting in mitral regurgitation
2. Left ventricular dilatation leading to failed mitral leaflet coaptation
3. Ventricular septal rupture
4. Free wall rupture
5. Ascending aortic dissection involving coronaries ± aortic valve
Causes of CS not related to AMI
1. Fulminant myocarditis
2. Hypertrophic cardiomyopathy with outflow obstruction
3. Decompensated dilated/restrictive cardiomyopathy
4. Tako‐tsubo cardiomyopathy
5. Peripartum/post‐partm‐cardiomyopathy
6. Post cardiotomy
7. Significant pulmonary embolism
8. Myocardial dysfunction related to neurological cause, for example, subarachnoid hemorrhage
9. Cardiac tamponade
10. Mitral stenosis

**Table 3:** Risk factors associated with development of cardiogenic shock

1. Older age
2. Female sex
3. Prior myocardial infarction (MI) or diagnosis of heart failure
4. History of hypertension and/or diabetes mellitus
5. Anterior ST elevation MI
6. Completed infarct
7. Multi‐vessel coronary artery disease
8. Complete heart block

## 4 INITIAL ASSESSMENT

CS is a medical emergency; a high index of suspicion, rapid diagnosis, and immediate commencement of treatment, including transferring patients to a tertiary cardiac center may influence clinical outcomes.^24^ Moreover, CS can develop at any time throughout the patient's illness, mainly following arrival to the hospital.^25^ History of chest pain and electrocardiographic changes of AMI with signs of CS will confirm the diagnosis. In addition, major non‐cardiogenic categories of shock, such as distributive shock (septic, neurogenic), hypovolemic shock (hemorrhage, dehydration), or obstructive shock (pulmonary embolism, pericardial tamponade, aortic dissection) should also be excluded, if clinically indicated. Physical examination is important in recognizing the features of hypoperfusion, as well as detecting mechanical complications. A chest X‐ray may be helpful in confirming the diagnosis of pulmonary edema. However, absence of pulmonary edema does not rule out CS. Mortality rates are similar in patients presenting with CS, irrespective of pulmonary edema.^26^ A bedside echocardiogram may provide highly valuable information, especially LV ejection fraction and severity of mitral regurgitation, as they are independent predictors of mortality in patients with CS.^6^ General measures, such as arterial oxygenation and near‐normal pH should be maintained. Some patients may require endotracheal intubation and mechanical ventilation.

Patients presenting with CS due to predominant RV failure are generally treated with aggressive fluid resuscitation with an intention to increase RV filling pressure. Such a practice may have limited role, as volume‐loading augments pulmonary capillary wedge pressure without improving cardiac index, aortic pressure or right/left ventricular stroke work index.^27^ In addition, the majority of these patients have elevated RV EDP, and any further increase may have detrimental effects.^4^ Although the routine use of right heart catheterization in managing critically ill patients in intensive care has declined, as their use was reported to be associated with higher mortality, and excess resource utilization.^28^ However, the Swan‐Ganz catheter may play a role in managing patients with predominant RV failure, as the cardiac output is higher (with/without inotropic support), when the RV EDP is between 10 and 15 mm Hg.^29^ Alternatively, non‐invasively assessed mitral deceleration time of less than 140 ms is predictive of pulmonary capillary wedge pressure of >20 mm Hg.^11^ Previously published articles have described in‐depth assessment of patients presenting with AMI‐CS.^3^, ^30^

## 5 GENERAL APPROACH

The most effective therapeutic intervention in patients with AMI complicated by CS (AMI‐CS) is establishment of coronary reperfusion, at the earliest possible. However, in the interim, their hemodynamic instability should be managed ensuring adequate oxygenation and ventilation, preservation of euvolemic state, and general critical care measures.

## 6 PHARMACOTHERAPY

Aspirin, heparin, and other pharmacotherapeutic agents should be used in accordance with the guidelines in managing patients presenting with AMI, as they are associated with better survival. Although, β‐blocker use reduces mortality in patients with AMI, caution should be exerted, as intravenous metoprolol use in the COMMIT trial was associated with increased incidence of CS, especially in first 24 hours from AMI,^31^ whereas in the SWEDEHEART registry, it was associated with excess in‐hospital CS and 30‐day mortality.^32^ Systemic hypoperfusion, a characteristic of CS, results in lactic acidosis that inhibits myocardial contractility. The early treatment of CS is aimed at preserving or restoring adequate CO to maintain tissue perfusion.

### 6.1 Supportive therapy

Administration of oxygen should be reserved for hypoxic patients as supplementary oxygen therapy increases coronary vascular resistance,^33^ and there are suggestions that its use in non‐hypoxic patient is associated with higher mortality.^34^

### 6.2 Inotropes and vasopressors

Inotropes and vasopressors are used in the management of patients with CS due to their favorable hemodynamic effect. They improve CO and tissue perfusion by increasing myocardial contractility and systemic vascular tone, respectively. With use of such sympathomimetic agents, focus should be to keep the doses to minimum possible; as they have deleterious effects at the cellular level that lead to excess mortality.^35^ Limited evidence exists with respect to the effectiveness of inotropes and vasopressors in CS.^36^

Norepinephrine use may be beneficial over dopamine, and should be the first drug of choice in hypotensive patients. The Sepsis Occurrence in Acutely Ill Patients (SOAP‐II) trial compared norepinephrine with dopamine as a first‐line agent in treating patients with shock of different etiologies. This study included 1679 patients, of whom 280 had CS. In the overall cohort, similar mortality was observed in both treatment arms; however, dopamine use was associated with more adverse events. Pre‐specified subgroup analysis revealed norepinephrine to be particularly beneficial in patients in cardiogenic shock.^37^ Therefore, the European Society of Cardiology (ESC) guidelines for management of STEMI complicated by CS recommend that norepinephrine should be preferred over dopamine when blood pressure is low (Class IIb, Level of evidence B).^38^ In another prospective study, evaluating therapies in CS patients, combination of norepinephrine‐dobutamine demonstrated similar improvement in hemodynamic parameters to norepinephrine alone.^39^ However, combination therapy group demonstrated significantly less lactic acidosis, lower heart rate, less arrhythmia, and reduced compromise in gastric mucosal perfusion.^39^ In eight of the mechanically ventilated CS patients, isolated dopamine use was associated with increased oxygen consumption, whereas combination of moderate doses of dobutamine and dopamine (7.5 μg/kg/min each), increased mean arterial pressure, and maintained pulmonary capillary wedge pressure within normal limits.^40^ Epinephrine use is associated with tachycardia, lactic acidosis, and a pro‐thrombotic milieu, whereas norepinephrine has anti‐thrombotic characteristics.^41^

Vasopressin is another agent utilized in many centers as a second‐line therapy. It is an endogenous vasopressor stored mainly in the posterior lobe of the pituitary gland and myocardium. Vasopressin releases in response to increased plasma osmolality, hypotension, and elevated wall stress.^42^, ^43^ In a retrospective analysis, comparing vasopressin and norepinephrine treatment in patients presenting with CS complicating AMI, vasopressin therapy increased mean arterial pressure without adversely affecting pulmonary capillary wedge pressure, urine output or other inotropic requirement.^44^ In a prospective randomized study, comparing vasopressin and norepinephrine vs norepinephrine alone in the treatment of catecholamine‐resistant vasodilatory shock, combination infusion proved to be superior to norepinephrine alone.^45^

Levosimendan is a calcium‐sensitizing agent that increases myocardial contractility without affecting intracellular calcium levels, so that ventricular diastolic relaxation is well preserved**. In** addition, levosimendan induces vasodilation resulting in reduced afterload. This synergistic combination of improved myocardial contractility and vasodilatation may results in efficient myocardial energy utilization. In a randomized, double‐blinded study evaluating the safety and efficacy in patients presenting with CS due to AMI, levosimendan reduced mortality in comparison with placebo without inducing hypotension or cardiac ischemia; however, patients with systolic blood pressure <90 mm Hg were excluded.^46^ Prophylactic levosimendan infusion in patients with LV ejection fraction of 35% or less, undergoing cardiac surgery did not reduce composite end point of death, renal‐replacement therapy, perioperative myocardial infarction, or mechanical assist device.^47^ Although, levosimendan is licensed in many countries worldwide; the FDA has not approved its use in the United States yet. Overall, majority of these agents improve myocardial contractility and CO, so that their use over a shorter period in patients with CS can be justified. Evidence supports combining these agents in lower doses, rather than using them at higher doses in isolation. Mechanisms, doses, and side effects of various agents are described in Table 4.

**Table 4:** Inotropes and vasopressors

		Mechanism of action				
Drug	Dose range	*α*	*β*1	*β*2	DA	Side effects
Dobutamine	2.0‐20.0 μg/kg/min(up to 40 μg/kg/min)	+	+++++	+++	NA	Tachycardia
						Ventricular arrhythmia
						Cardiac ischemia
						Hypertension (those on non‐selective β‐blocker)
Dopamine	2.0‐20.0 μg/kg/min	+++	++++	++	+++++	Tachycardia
						Ventricular arrhythmia
	(Up to 50 μg/kg/min)					
						Cardiac ischemia
						Tissue ischemia/gangrene
						Hypertension (those on non‐selective β‐blocker)
Norepinephrine	0.01‐3 μg/kg/min	+++++	+++	++	NA	Atrial/ventricular arrhythmia
						Tissue ischemia
						Hypertension (those on non‐selective β‐blocker)

Epinephrine	0.01‐0.1 μg/kg/min	+++++	++++	+++	NA	Ventricular arrhythmia
						Cardiac ischemia
						Hypertension
						Sudden cardiac death
Vasopressin	0.01‐0.1 U/min (Bolus: 40 U)	Dose dependent increase in systemic vascular resistance and vagal tone				Arrhythmia Hypotension
		Increases vascular sensitivity to norepinephrine				Cardiac ischemia
		V_1a_: Constriction of vascular smooth muscle				Splanchnic vasoconstriction
		V_2_: water reabsorption (renal collecting duct)				Tissue ischemia
Levosimendan	0.05‐0 .2 μg/kg/min (Loading: 12‐24 μg/kg over 10 minutes)	Calcium sensitization of contractile proteins (myocytes)(Improves myocardial contractility without increasing intracytosolic Ca^++^)				Tachycardia
						Hypotension
						Enhanced AV conduction
		Opening of ATP‐dependent K^+^ channels (vascular smooth muscle)				
		(Vasodilatation results in reduced afterload)				

## 7 CORONARY REVASCULARIZATION

Emergent revascularization to restore myocardial blood supply in patients with AMI‐CS has consistently demonstrated to offer mortality benefit.^8^, ^48^ Therefore, the priority should be to transfer them to a center with PCI and surgical revascularization capabilities. The SHOCK trial demonstrated the importance of early revascularization either by PCI or coronary artery bypass surgery (CABG) in patients with AMI complicated by CS. The study demonstrated mortality benefit at 6 months, extending up to 6 years.^48^ Current ACC/AHA guidelines recommend primary PCI in patients presenting with STEMI complicated by CS, irrespective of age (Class I recommendation), but no specific recommendation were made in regards to PCI in patients with AMI‐CS.^49^ Whereas, the initial 2017 ESC guidelines recommend PCI of all high‐grade lesions in such patients before hospital discharge.^50^ However, later published results from the culprit lesion only PCI vs multi‐vessel PCI in CS (CULPRIT‐SHOCK), a multicenter, randomized, open‐label trial comparing mult‐ivessel vs IRA‐only PCI, in patients presenting with AMI‐CS, demonstrated that PCI to an IRA only, resulted in lower death and need for renal replacement therapy at 30 days, whereas mortality was not different at 12 months between both groups.^51^ In light of this robust evidence from the CULPRIT‐SHOCK trial, the ESC revised their initial recommendation and proposed that PCI should be restricted to IRA only.^52^ Immediate multi‐vessel PCI should be offered only when it is difficult to identify IRA or there are multiple culprit lesions. Staged PCI to a non‐IRA should be based upon risks and benefits associated with a new procedure.^52^

## 8 MECHANICAL CIRCULATORY SUPPORT DEVICES

Over the last two decades, we have seen the introduction and increased utilization of various mechanical circulatory support (MCS) devices that can offer hemodynamic support, independent of myocardial contractility.^53^ In general, MCS can be classified as those to be used for short vs long‐term, deployed percutaneously vs surgically, or based upon their mechanisms.

### 8.1 Intra‐aortic balloon pump

For years, the Intra‐aortic balloon pump (IABP) served as the mainstay MCS therapy for patients presenting with AMI‐CS. IABP augmented coronary and peripheral perfusion, and increasing CO by 0.5 L/min.^54^ In the SHOCK trial, patients who demonstrated hemodynamic improvement with IABP use had better survival.^55^ However, in a prospective randomized multi‐center IABP SHOCK‐II study, IABP use failed to demonstrate any benefit, including hemodynamic stabilization, length of stay in the intensive care unit, need for inotropic support, and most importantly, mortality.^56^ Evidences supporting use of IABP in AMI‐CS patients is very week, as observed in the latest ACC/AHA (class IIa, level of evidence B),^57^ and the ESC guidelines (class IIb, level of evidence B).

### 8.2 Impella

An early attempt at using a catheter‐mounted, axial flow device positioned across the aortic valve to offer hemodynamic support in CS patients was performed nearly 20 years ago.^58^ Impella (Abiomed Inc., Danvers, Massachusetts) works on the same principle. The Impella family includes devices capable of augmenting circulatory support by 2.5, 3.5, and 5.0 L/min. The Impella 2.5 has a 12 Fr. pump motor size, and can be inserted through a 13 Fr. sheath. Similarly, the Impella CP, which offers circulatory support up to 4 L/min can be inserted through a 14 Fr. sheath, whereas Impella 5.0 requires surgical cut‐down. In the ISAR‐SHOCK trial comparing efficacy of Impella 2.5 vs IABP in patients presenting with CS (25 patients in total), the Impella 2.5 offered superior hemodynamic support compared with the IABP; however, it failed to demonstrate a 30‐day mortality benefit, and was associated with a higher incidence of hemolysis.^59^ In a retrospective multi‐center Impella‐EURO SHOCK registry of 120 patients, Impella 2.5 use demonstrated improved hemodynamic parameters and better organ perfusion.^60^ The USpella registry demonstrated better survival and more complete revascularization in patients presenting in CS, who had an Impella 2.5 implanted pre‐PCI.^61^ The on‐going National CS Initiative will be the seminal trial of this device. Initial data demonstrated improved mortality with Impella over historical data from the SHOCK study.^62^ The advantages of the Impella devices are familiar implantation technique (similar to pigtail catheter), and single arterial access. Even though the Impella offers superior hemodynamic performance than the IABP, no mortality benefit has been demonstrated thus far. At the same time, this reliable hemodynamic profile comes at the cost of increased risk of vascular complications.^63^, ^64^ The FDA has approved all Impella devices for partial circulatory support for up to 6 hours.

### 8.3 TandemHeart

The TandemHeart (CardiacAssist, Inc., Pittsburgh, Pennsylvania) is another peripheral MCS that can provide 3.5 to 4.5 L/min of flow. The TandemHeart offered superior hemodynamic support than the IABP, but also failed to demonstrate a 30‐day mortality benefit.^65^ In patients with CS that was refractory to IABP and vasopressor support, deployment of the TandemHeart was associated with rapid improvement in hemodynamic and metabolic parameters.^66^ The TandemHeart can also offer hemodynamic support in patients with RV failure.^67^ As TandemHeart insertion requires transseptal catheter placement in the left atrium, an operator requires skills to perform a septal puncture. In addition, its use is associated with higher bleeding and ischemic limb complications.^68^ The FDA has approved the device for circulatory support for up to 6 hours. Various MCS devices are summarized in Table 5.

**Table 5:** Percutaneous mechanical circulatory support devices

	Mechanism	Insertion/size	Support offered	Complication	Difficulty of insertion	Cost
IABP	Pneumatic	Femoral artery 7‐9 F	0.5 L/min	Bleeding	+	+
				Limb ischemia		
				Vascular complication		
Impella	Axial	Femoral artery	2.5/3.5/5.0 L/min	Hemolysis	++	++
				Bleeding		
		2.5‐12 F		Limb ischemia		
		CP‐14 F				
		5.0‐22 F				
Tandem‐Heart	Centrifugal	21 F—Left atrium (outflow)	3.5‐4.5 L/min	Limb ischemia	++++	+++
				Bleeding		
		Requires trans‐septal puncture				
				Vascular complication		
				Hemolysis		
		15‐17 F— Femoral artery (outflow)				
ECMO	Centrifugal	18‐31 F— right atrium (inflow)	>4.5 L/min	Limb ischemia	++	+++
				Hemolysis		
				Stroke		
		15‐22 F— Femoral artery (outflow)				
				Bleeding		
HeartMate percutaneous heart pump (PHP)	Axial	14 F	4‐5 L/min	Limb ischemia	++	++
				Bleeding		
				Vascular complication		
				Hemolysis		

### 8.4 Newer devices

In addition to the aforementioned devices, various other peripheral MCS devices are under development. The Reitan catheter pump (Kiwimed, London, UK) is a catheter mounted foldable propeller, capable of providing circulatory support of up to 5 L/min. The catheter is positioned in the descending aorta, where it works in a series with heart and reduces left ventricular afterload.^69^ Safety and efficacy of its use in acutely decompensated heart failure patients has been verified.^70^ Similarly, the iVAC 2 L and 3 L (PulseCath BV, Amsterdam, Netherlands) is a 17‐21F catheter with an integrated 2‐way valve system, capable of offering circulatory support of 2 to 3 L/min. Standard IABP console can also drive an MCS from PulseCath. In addition, this MCS device can also be used as a ventricular assist device (VAD) to support the right ventricle, which requires insertion through the pulmonary artery.^71^

### 8.5 Extracorporeal membrane oxygenation

Although this technology was introduced more than five decades ago, availability of smaller cannulas as well as lightweight portable consoles has resulted in its resurgence. The extracorporeal membrane oxygenation (ECMO) can be used in two configurations: veno‐venous ECMO (VV‐ECMO) and veno‐arterial ECMO (VA‐ECMO). VV‐ECMO is used only for respiratory failure, whereas VA‐ECMO offers cardiac and pulmonary support. CS is one of the fastest growing indications for its use.^72^ ECMO offers flow rates of 3 to 4 L/min. A wealth of evidence supporting ECMO as MCS comes from experience with treating post‐cardiotomy CS,^73^ AMI‐CS or assisting high‐risk PCI. ECMO‐assisted PCI was shown to be an independent predictor of 30‐day survival in patients presenting with AMI complicated with profound CS.^74^ However, authors stated that patients were enrolled on a contemporary basis and therefore, there may be an impact of non‐identical treatment on outcomes in the two groups.^74^ Portable ECMO support can be initiated in patients with refractory CS; either in the out‐of‐hospital setting or in the referring hospital, even in situation of ongoing cardio‐pulmonary resuscitation and these patients can be transferred for further care in a tertiary center or to the catheterization laboratory for PCI.^75^ In a series of patients treated with ECMO support for CS, 42% survived to hospital discharge. However, 57% of patients suffered ECMO‐related major complications,^76^ such as lower limb ischemia, requirement for amputation, compartment syndrome with a potential need of fasciotomy, stroke, major bleeding, renal failure, and device‐related infections.^77^ Similar to other MCS devices, the timing of ECMO support initiation remains unclear; however, once the features of end‐organ damage have developed, mortality remains very high, in spite of ECMO use.^76^ There are no randomized trials demonstrating mortality benefit of ECMO in patients with CS. Despite these benefits, the use of ECMO is limited by need for adequately sized peripheral vasculature, requirement of perfusionist, and short support time.^38^ Although ECMO reduces blood‐flow and strain on heart, such mechanical support augments systemic arterial afterload; concomitant use of impella or possibly IABP along with ECMO may offer solution to such hemodynamic alteration.^78^ Ongoing National CS Initiative (ClinicalTrial.Gov; NCT03677180) will be the seminal trial of MCS. However, the preliminary results demonstrated improved mortality with Impella, if the support was initiated early after shock onset, before initiation of inotropes or vasopressor and before PCI.^79^ Based upon the RECOVER RIGHT, an Investigational Device Exemption study demonstrating 44/60 (73.3%) patients surviving 30‐days, the FDA approved Impella right percutaneous (RP) system to support the failing right ventricle, whereas recently the FDA has issues a letter to healthcare providers, making them aware of observed excess mortality in the ongoing post‐approval study.

An ideal peripheral MCS device should be the one that provides effective and reliable circulatory support, easy and quick to insert (preferably percutaneously), easy to operate and maintain following insertion, and is associated with a low rate of complications. Most importantly, such a device should offer mortality benefit in addition to improved hemodynamic parameters.

### 8.6 Surgically implanted ventricular assist device

Peripheral MCS devices offer adequate temporary circulatory support that may be enough to break the downward spiral course of hemodynamic compromise. However, some patients require long‐term and complete circulatory support that can be achieved with surgically implanted ventricular assist device (SVADs). The Abiomed BVS 5000 (Abiomed, Inc., Danvers, Massachusetts) was the earliest example of a SVAD, first attempted in a patient in 1990. In an observational study, Abiomed BVS 5000 demonstrated its effectiveness as a bridge‐to‐recovery or bridge‐to‐transplantation in patients presenting with ventricular failure.^80^ This SVAD is an external, pneumatically driven device that requires insertion of an inflow cannula into the left atrium, and an outflow cannula into the aorta (LV assist). Similarly, this device can also be used to drain blood from the right atrium and return it to pulmonary artery to function as a RVAD. CentriMag VAD (Levitronix, Waltham, Massachusetts) is a magnetically levitated, centrifugal continuous flow pump.^81^ The FDA has approved CentriMag for circulatory support for up to 6 hours, and under “humanitarian use approval” for up to 30 days. Despite development of various SVADs to offer significant circulatory support, mortality benefit is yet to be reported. At present, few centers are capable of offering SVADs services. In addition, need for general anesthesia, and issues with availability of operating rooms as well as surgeons are some of the limiting factors with SVAD use. Moreover, it is an open surgical procedure that is also associated with bleeding, infection, and ischemic complications. Patients without recovery of myocardial function may be advanced to permanent surgically‐implanted LVADs, namely HeartMate II, HeartMate III, HeartWare, or SynCardia.

### 8.7 Mechanical complications

Mechanical complications of AMI, such as rupture of the ventricular septum (VSR), free wall or a papillary muscle result in hemodynamic instability, high mortality, and pose a significant challenge as far as the management is concerned.^22^, ^82^ A mechanical complication should be suspected in the event of rapid change in hemodynamic parameters.^83^ Risk of mechanical complications was higher in the thrombolytic era compared with the current era with primary PCI as the standard of care.^23^, ^80^ Patients with VSR may be clinically stable in the early period but have a variable course, with a grim long‐term outcome, especially in the elderly and those with poor RV function.^83^ Surgical repair of ventricular septal rupture is challenging, especially deploying sutures in the necrotic myocardium. However, external septal plication for VSR, and Gore‐Tex patch repair of free wall rupture have been reported.^84^, ^85^ An attempt can be made at percutaneous repair of VSR by deploying a septal occluder; however, choosing the appropriate size is a challenge, as the size of the post‐infarct VSR may increase with time.^86^ Similarly, acute mitral regurgitation (MR) is not uncommon in the setting of an AMI, and can cause or exacerbate CS. Acute MR can be due to chordal or papillary muscle rupture, papillary muscle dysfunction or left ventricular dilatation leading to poor coaptation of the valve leaflets. Even though no randomized studies have been conducted, patients who develop ischemic MR do better with CABG and MV repair or replacement than with PCI alone.^87^

### 8.8 Mortality and long‐term outcome

Shock is associated with high in‐hospital and 30‐day mortality, but if survived the initial insult, these patients have overall better quality of life and longevity.^48^, ^88^ Follow‐up data from the SHOCK trial reported 32.8% 6‐year survival in early revascularization group, with 13.2% absolute difference in comparison with patients managed by medical stabilization.^48^ Importantly, the medically managed patient cohort demonstrated disproportionately high mortality in the first year following CS (26.4/100 patient‐years vs 9.5/100 patient‐years). After the first year, annualized death rates were 8.0 and 10.7/100 patient‐years in the revascularization and conservative stabilization groups, respectively.^48^ Eleven year follow‐up data from the US patients, who participated in the Global Utilization of Streptokinase and Tissue‐Type Plasminogen Activator for Occluded Coronary Artery (GUSTO)‐1 trial, reported 2% to 4% yearly mortality irrespective of their presentation with or without shock.^88^ In a prospectively collected registry of patients with AMI‐CS, 80% of in‐hospital survivors were in New York Heart Association Functional Classification (NYHA) class I/II at a median follow‐up of 18.1 months.^89^ Increasing age, female sex, baseline renal dysfunction, long‐time from symptom onset to revascularization, and thrombolysis in myocardial infarction (TIMI) flow less than 3 at the end of PCI, are factors associated with high mortality in CS patients.

Attempts are made at various front to improve the outcomes in patients with AMI complicated by CS. Recently published articles have proposed the need for team‐based approach in managing this patient population; especially establishing advanced cardiac shock care centers.^90^, ^91^ In addition to accepted “door‐to‐balloon time,” there is emerging concept of “door‐to‐support/unloading time” using mechanical circulatory support in patients with AMI‐CS.^92^ Adopting a regional shock protocol, the “Detroit cardiogenic shock initiative” has demonstrated the feasibility and effectiveness of establishing early mechanical circulatory support in AMI‐CS patients.^93^ On the contrary, ideal timing of initiating mechanical circulatory support, and its mortality benefit; effectiveness of recently published ORBI risk score in identifying patients at risk of developing CS after presentation with AMI,^94^ and effectiveness of therapeutic hypothermia in patients with CS^81^ warrants further evaluation.

## 9 CONCLUSION

With early revascularization, the frequency of CS complicating AMI, and resultant mortality have reduced, albeit overall mortality still remains very high, and treatment options are limited. Early diagnosis and institution of therapy to break the vicious circle of LV dysfunction, and resultant coronary/tissue hypoperfusion are of paramount importance. Although MCS/VADs significantly improve hemodynamic parameters and end‐organ perfusion in patients with CS, till date such support devices have failed to demonstrate mortality benefit. There is clearly a need for further randomized trials to assess newer drugs, support devices, and treatment strategies.

## CONFLICT OF INTEREST

The authors declare no potential conflict of interests.
